# Effects of Reduced Crude Protein Diets with Graded Methionine Supplementation on Growth Performance, Nitrogen Utilization, and Serum Metabolomic Profiles in Growing–Finishing Pigs

**DOI:** 10.3390/ani16111687

**Published:** 2026-05-31

**Authors:** Seong Hoon Shim, Su Hyun An, Minji Kim, Jinyoung Jeong, Hwan Ku Kang

**Affiliations:** Precision Animal Nutrition Division, National Institute of Animal Science, Wanju 55365, Republic of Korea; kanji0616@korea.kr (S.H.S.);

**Keywords:** low crude protein, methionine, nitrogen utilization, serum metabolomics

## Abstract

This study evaluated reduced dietary crude protein (CP) with graded methionine (Met) supplementation in growing and finishing pigs. In each phase, 48 crossbred barrows were assigned to four treatments with four replicate pens per treatment and three pigs per pen. The growing and finishing trials lasted 43 and 50 d, respectively. Growing pigs received CON (16% CP, 0.37% Met) or low-CP diets (15% CP) with 0.37%, 0.41%, or 0.44% Met; finishing pigs received CON (14% CP, 0.27% Met) or low-CP diets (13% CP) with 0.27%, 0.30%, or 0.32% Met. Nitrogen excretion and apparent total tract digestibility of nitrogen were assessed through metabolism trials. Serum biochemical parameters were analyzed at the end of each phase, and untargeted ^1^H-NMR-based serum metabolomic analysis was performed in finishing pigs. Most growth performance variables, nitrogen excretion, and nitrogen digestibility were not significantly affected by treatment, whereas selected blood urea nitrogen concentrations were reduced in low-CP diets with Met supplementation. Serum metabolomic profiling suggested exploratory shifts in energy-related metabolites. Overall, these findings suggest that a one-percentage-point reduction in dietary CP with Met supplementation can maintain overall pig performance while inducing selected nitrogen-related biochemical and metabolic responses under the conditions of this study.

## Simple Summary

Reducing the protein content of pig feed is a nutritional strategy for improving the sustainability of pork production. However, excessive protein reduction may negatively affect pig growth and health, so the balance of essential nutrients must be carefully considered. This study evaluated whether a diet with one percentage point less protein, combined with graded supplementation of methionine, an essential nutrient required for growth and metabolism, could be used for growing and finishing pigs. The reduced-protein diets with methionine supplementation generally maintained body weight gain, feed intake, feed efficiency, carcass traits, and meat quality under the conditions of this study. Nitrogen losses in feces and urine were not clearly reduced, but some blood measurements related to protein use were changed. In finishing pigs, the analysis of small molecules in blood suggested possible changes in energy-related metabolic pathways. These results indicate that a modest reduction in dietary protein, when properly balanced with methionine, can maintain overall pig performance and may support the development of more efficient feeding strategies.

## 1. Introduction

Per capita meat consumption in OECD countries increased by ~1.4% in 2025 compared with 2024 [1], suggesting sustained consumer demand for meat products. However, the expansion of meat consumption is accompanied by increasing environmental burdens associated with livestock production systems. In particular, nitrogen (N) excreted in pig manure is a major contributor to environmental pollution, since excessive N loading can contaminate soil and water resources and exacerbate eutrophication in aquatic ecosystems. Manure-derived N can be lost as reactive N forms, such as ammonia (NH_3_), contributing to air quality deterioration and secondary environmental pollution [2]. Accordingly, mitigating manure-based N output has become an important priority in swine production, and several management and nutritional approaches have been investigated to reduce N losses at the farm level.

Lowering dietary crude protein (CP) while supplementing limiting amino acids to meet requirements has been widely adopted to reduce N excretion and maintain amino acid balance in monogastric diets [3]. A decrease in dietary CP, combined with appropriate amino acid supplementation, decreases total dietary N intake, which can reduce the proportion of absorbed amino acids that are catabolized and excreted. Consequently, the formation of ammonia (NH_3_) during amino acid transamination and oxidative deamination in the liver is reduced, leading to decreased urea synthesis via the urea cycle and lower urinary urea N excretion [4]. Similarly, recent evidence from low-CP diet studies indicates that reducing dietary CP by 1% while balancing limiting amino acids can decrease manure N excretion by ~8.4%, supporting the potential of low-CP feed for mitigating manure-based N output [5,6]. However, excessive reductions in dietary CP may restrict essential and nonessential amino acid supply, disrupt amino acid balance, and impair productivity, including average daily gain (ADG) and gain-to-feed ratio (G:F). Therefore, previous studies evaluated whether pig performance can be maintained under lower-CP feeding with supplementation of limiting amino acids according to the ideal protein concept. For example, a stepwise reduction in CP from 15.16% to 13.50% with adequate balancing of limiting amino acids maintained growth rate and feed efficiency and decreased blood urea nitrogen (BUN) and fecal and urinary N outputs in late-finishing pigs [6]. Similarly, reducing dietary CP by ~2% throughout the growing–finishing period (25–125 kg body weight) and balancing essential amino acids reduced N excretion without compromising growth performance or carcass traits [7].

Responses to low-CP diets have not been fully consistent across studies; even within a given experiment, outcomes may vary depending on factors such as dietary energy level, ambient temperature, and genetic background [8]. Therefore, the applicability and effectiveness of low-CP diets should be interpreted by considering not only the absolute CP level but also the growth stage, ingredient composition, energy-to-amino acid balance, and environmental conditions. Previous studies on the effects of low-CP diets have largely focused on production indicators (growth performance, carcass traits, and N excretion), while neglecting metabolic and physiological parameters, including serum biochemistry and the metabolome.

Therefore, this study aimed to evaluate whether a modest one-percentage-point reduction in dietary crude protein, combined with graded methionine supplementation, could maintain growth performance and carcass traits while modifying nitrogen utilization and metabolic responses in growing and finishing pigs. In addition, serum ^1^H-NMR-based metabolomic profiling was used as an exploratory approach to identify potential metabolic alterations associated with low-CP diets and Met supplementation. We hypothesized that a modest one-percentage-point reduction in dietary CP, when combined with graded Met supplementation, would maintain growth performance and carcass traits while modulating nitrogen-related biochemical responses and circulating metabolite patterns. The findings of this study are expected to provide useful information for understanding the productive and nitrogen metabolism-related physiological responses of pigs to reduced dietary CP with graded Met supplementation.

## 2. Materials and Methods

### 2.1. Animals, Feed, and Experimental Design

Experimental diets were formulated according to the nutrient requirements for growing and finishing pigs recommended by the National Research Council [9]. Crossbred barrows (Landrace × Yorkshire × Duroc) were used in both experiments.

The metabolism trials were conducted using separate sets of barrows that were not included in the main feeding trials. The initial body weights used in the growing- and finishing-phase metabolism trials were 61.93 ± 0.82 and 87.77 ± 0.39 kg, respectively. Pigs used in the metabolism trials were individually housed in metabolic crates and fed 3% of average body weight in two equal meals per day, with free access to water [10].

In the growing feeding trial, pigs with an initial body weight of 32.50 ± 1.05 kg were assigned one of four dietary treatments: a control diet containing 16% CP and 0.37% Met (CON) and three low-CP diets containing 15% CP with graded Met levels [T1 (0.37% Met), T2 (0.41% Met), and T3 (0.44% Met)]. In the finishing feeding trial, pigs with an initial body weight of 58.75 ± 1.72 kg were assigned a control diet containing 14% CP and 0.27% Met or low-CP diets containing 13% CP with graded Met levels [T1 (0.27% Met), T2 (0.30% Met), and T3 (0.32% Met)]. The growing and finishing feeding trials lasted 43 d (day 0 to day 43) and 50 d (day 0 to day 50), respectively. Both feeding trials used a randomized complete block design, in which pigs were blocked according to initial body weight before assignment to dietary treatments. Each treatment had four replicate pens with three pigs per pen, resulting in 48 pigs per phase. The number of experimental units per treatment was determined before the experiment using the Animal Experiment Sample Size Calculator (AEEC), with reference to the statistical power considerations for swine nutrition experiments described by Aaron and Hays [11]. Under the assumptions used in the calculator, four experimental units per treatment were considered sufficient to detect a significant treatment difference at α = 0.05. For growth performance, the pen was considered the experimental unit. Feed and water were provided ad libitum. Pigs were housed in environmentally controlled pens maintained at 25 ± 2 °C during the growing phase and 22 ± 2 °C during the finishing phase, with relative humidity maintained at 60 ± 10%. The ingredients and nutrient compositions of the experimental diets are presented in Table 1 and Table 2.

Separate metabolism trials were conducted during both the growing and finishing phases to evaluate nitrogen excretion. For each phase, 12 barrows with body weights close to the mean body weight of the corresponding phase were randomly selected and assigned to a two-period balanced incomplete Latin square arrangement with four dietary treatments and three replicate squares. Each period lasted 7 d and consisted of a 4-d adaptation period followed by a 3-d total collection period for feces and urine. Each treatment was represented by three pigs in each period, resulting in three observations per treatment per period and six observations per treatment across the two periods. The individual pig was considered the experimental unit.

The treatment allocation was generated using a spreadsheet-based Latin square allocation program described by Kim and Kim [12]. The allocation was arranged so that each treatment appeared equally within each period and no pig received the same treatment in both periods. The program-generated balanced sequence and the 4-d adaptation period before sample collection were used to limit potential carryover effects, although residual carryover effects could not be independently estimated in the two-period design. The detailed treatment allocation matrix for the metabolism trials is provided in Supplementary Table S1.

### 2.2. Growth Performance Evaluation

Initial and final body weights were recorded to calculate the ADG, average daily feed intake (ADFI), and G:F. ADG was calculated as the total body weight gain divided by the number of experimental days. ADFI was expressed as the average daily feed intake per pig (kg/pig/d). Feed efficiency was calculated as ADG/ADFI. During the metabolism trial, ADG, ADFI, and G:F were determined in the same manner over each 7-d period.

### 2.3. Nutrient Digestibility and Nitrogen Excretion

Fecal and urinary samples collected during the metabolism trials were used to determine nutrient digestibility and nitrogen excretion. Fecal samples were dried in a forced-air oven at 65 °C for 96 h, ground through a 1-mm screen using a Wiley mill, and stored until analysis. Urine samples were collected using funnels installed beneath the metabolic crates, and 10 mL of 6 N HCl was added per liter of urine immediately after collection to prevent ammonia volatilization. Fecal and urinary samples were stored at −20 °C until analysis. The dry matter (method 930.15) and nitrogen (method 990.03) contents of the fecal samples were determined according to AOAC [13]. The apparent total tract digestibility (ATTD) of nitrogen was calculated as follows: ATTD (%) = (nitrogen intake − fecal nitrogen output)/nitrogen intake × 100. Nitrogen retention was calculated as nitrogen intake minus fecal and urinary nitrogen output. Biological value (BV) was calculated as follows: BV (%) = retained N/(N intake − fecal N output) × 100.

### 2.4. Carcass Traits and Meat Quality

At the end of the finishing feeding trial, five pigs per treatment were selected for carcass and meat quality analysis. The pigs were selected from the corresponding treatment groups by considering final body weight close to the treatment mean and by excluding pigs with abnormal health conditions. Pen representation was considered during selection to reduce potential pen-level selection bias. Live body weight was measured immediately before shipment using a weighing scale (WA-400, Meier-Brakenberg, Extertal, Germany). After 2 h in lairage, the pigs were slaughtered at Naju Livestock Products Joint Market (NongHyup Agribusiness Group Inc., Naju, Republic of Korea), a licensed abattoir, following electrical stunning and exsanguination. After chilling at 0 °C for 18 h, the carcasses were cut between the 10th and 11th ribs, and longissimus dorsi samples were collected from the left side for further analysis. Backfat thickness and intramuscular fat thickness were measured on the sampling day and used for carcass yield calculations. Cooking loss was determined by heating loin samples (3 cm thick) in a water bath (WSB-45, Daihan Scientific, Wonju, Republic of Korea) at 80 °C until the core temperature reached 70 °C, followed by cooling under running water for 30 min. The proximate composition (moisture, crude fat, and crude protein) was analyzed according to AOAC [13] using near-infrared spectroscopy (FoodScan, Foss, Hillerod, Denmark). Each sample (~200 g) was ground, placed in a round Petri dish, and analyzed in triplicate. Meat color was measured in triplicate using a chromameter (CR-300, Konica Minolta, Tokyo, Japan) to determine the CIE L* and a* values.

### 2.5. Serum Biochemistry and Metabolomic Profiling

At the end of each feeding trial, blood samples were collected from four pigs per treatment on day 43 for the growing phase and day 50 for the finishing phase. For blood biochemical analysis, one pig was selected from each replicate pen within each treatment, considering body weight close to the pen or treatment mean and excluding pigs with abnormal health conditions. Blood samples were collected in the morning before routine feed replenishment, without prior fasting, via jugular venipuncture to minimize variation related to sampling time and feeding status. Briefly, 10 mL blood was collected into BD Vacutainer SST tubes, allowed to clot at room temperature, and centrifuged at 3000 rpm for 15 min (HA-12, Hanil Scientific, Gimpo, Republic of Korea) to obtain serum, which was stored at −80 °C. Serum biochemical parameters were analyzed using an automated dry-chemistry analyzer (Catalyst Dx, IDEXX Laboratories, Westbrook, ME, USA) with manufacturer-provided reagent slides according to the manufacturer’s instructions. Blood urea nitrogen (BUN), creatinine (CREA), total cholesterol (CHOL), and inorganic phosphorus (PHOS) were measured using the corresponding chemistry slides. Alanine aminotransferase (ALT) and gamma-glutamyl transferase (GGT) were determined using kinetic enzyme activity assays. Globulin (GLOB), BUN/CREA ratio, and albumin (ALB)/GLOB ratio were calculated from the measured biochemical values. For metabolomic analysis, the same serum samples collected from the four selected pigs per treatment at the end of the finishing phase were used. For ^1^H-NMR-based metabolomic analysis, serum samples were thawed at 4 °C and centrifuged at 12,500 rpm for 10 min at 4 °C to collect the supernatant. The serum supernatant (150 µL) was mixed with 450 µL of D_2_O-based phosphate buffer adjusted to pH 7.4 ± 0.1, giving a serum-to-buffer ratio of 1:3, and transferred into 5-mm NMR tubes. For ERETIC-based quantitative scaling, pooled serum was prepared by collecting 20 µL from each sample, and the pooled serum supernatant (150 µL) was mixed with 438 µL of D_2_O-based saline solution and 12 µL of 100 mM valine solution to obtain a final valine concentration of 2 mM. ^1^H-NMR spectra were acquired on a Bruker AVANCE III HD 800 MHz spectrometer (Bruker BioSpin, Rheinstetten, Germany) using a Carr–Purcell–Meiboom–Gill pulse sequence (cpmgpr1d) to minimize broad signals originating from macromolecules. The acquisition parameters were as follows: relaxation delay, 2 s; dummy scans, 16; number of scans, 128; acquisition time, 2 s; time domain, 64k; receiver gain, 256; spectral width, 20 ppm; and sample temperature, 25 °C. All spectra were processed using Chenomx NMR Suite (v.10.1; Chenomx, Edmonton, AB, Canada). Baseline and phase were corrected using a Chenomx Processor, and the spectra were calibrated to the formate peak at 8.445 ppm. The formate peak was used only for chemical-shift calibration and was not used for quantitative normalization. The ERETIC signal was calibrated against the 2 mM valine standard and served as the defined quantitative reference for metabolite profiling. After ERETIC-based scaling, spectra were converted in Chenomx (v.10.1; Chenomx Inc., Edmonton, AB, Canada) using a DSS concentration setting of 0.5 mM. This DSS concentration setting was used only as a virtual software parameter for Chenomx-based profiling of the ERETIC-scaled spectra. DSS was not physically added to the serum samples and was not used as an independent quantitative reference. Thus, the quantitative output was traced to the ERETIC reference calibrated with the 2 mM valine standard. The spectral region of δ 0.2–10.0 ppm was used for downstream analysis. Metabolite identification and quantification were performed using the Chenomx Profiler and Livestock Metabolome Database. Each metabolite was assigned based on chemical shift, multiplicity, and spectral fitting using the Chenomx 800 MHz reference library. All fittings and residuals were manually inspected to confirm accurate peak assignments and exclude misidentified compounds.

### 2.6. Statistical Analysis

Data were analyzed using the MIXED procedure of SAS version 9.4 (SAS Institute Inc., Cary, NC, USA). For the feeding trials, treatment was included as a fixed effect and block as a random effect, with pen considered the experimental unit. For the metabolism trials, the incomplete Latin square design was analyzed with treatment and period as fixed effects and square and pig within square as random effects. Individual pig was considered the experimental unit for the metabolism trials. Least-squares means were compared using Tukey’s adjustment. Statistical significance was declared at *p* < 0.05, and 0.05 ≤ *p* < 0.10 was considered a tendency.

Linear and quadratic responses to graded methionine (Met) supplementation were evaluated using orthogonal polynomial contrasts among the low-crude protein (CP) treatments. The CON group was excluded from these contrasts because it differed in dietary CP level. Because Met levels were unequally spaced among the low-CP treatments, contrast coefficients were calculated based on the actual dietary Met concentrations within each phase. For the growing phase, the coefficients for T1, T2, and T3 were −11, 1, and 10 for the linear contrast and 3, −7, and 4 for the quadratic contrast. For the finishing phase, the coefficients for T1, T2, and T3 were −8, 1, and 7 for the linear contrast and 2, −5, and 3 for the quadratic contrast.

Serum metabolomic data were analyzed using MetaboAnalyst version 6.0. After median normalization and autoscaling, principal component analysis (PCA) and partial least squares–discriminant analysis (PLS-DA) were performed to explore treatment-associated patterns. Metabolites with variable importance in projection (VIP) scores ≥ 1.20 were considered potential contributors to the multivariate pattern. Individual metabolites were compared using one-way analysis of variance followed by Tukey’s post hoc test. Kyoto Encyclopedia of Genes and Genomes (KEGG) pathway analysis was performed using the criteria of −log10(*p*) ≥ 2 and pathway impact ≥ 0.2.

## 3. Results

### 3.1. Growth Performance of Growing Pigs

In the growing phase, the one-percentage-point CP reduction combined with graded Met supplementation did not significantly affect most growth performance variables during the 43-d feeding period, although ADG and G:F showed tendencies for an overall treatment effect (Table 3). Notably, the initial and final body weights were similar among the treatment groups (*p* = 0.660 and 0.278, respectively). The overall treatment effect for ADG tended to be significant (*p* = 0.093). Although ADG was numerically lower in the low-CP treatments than in the CON group, no clear dose-dependent response to increasing Met supplementation was observed. ADFI did not differ among the treatment groups (*p* = 0.631). The overall treatment effect for G:F also tended to be significant (*p* = 0.071), and Met supplementation exhibited a quadratic effect on G:F (*p* = 0.049), whereas no linear response was observed (*p* = 0.294).

### 3.2. Nitrogen Excretion and Digestibility in Growing Pigs

Growth performance was evaluated to confirm appropriate feeding during the metabolism trial (Table 4). Final body weight, ADG, and G:F did not differ among treatments (*p* > 0.05). However, ADG and G:F showed a tendency for a linear response to Met level among the low-CP treatments (*p* = 0.085), reflecting the numerical increase from T1 to T3 during the metabolism trial. In the growing phase metabolism trial, a one-percentage-point reduction in dietary CP combined with graded Met supplementation did not significantly change fecal nitrogen excretion (*p* = 0.792), urinary nitrogen excretion (*p* = 0.248), or ATTD of nitrogen (*p* = 0.749). Met supplementation did not exert any linear or quadratic effects on fecal nitrogen excretion and ATTD (*p* > 0.05). Although urinary nitrogen excretion, nitrogen retention, and biological value (BV) were not affected by the treatments (*p* > 0.05), Met supplementation tended to exert a linear effect on urinary nitrogen excretion (*p* = 0.063), nitrogen retention (*p* = 0.079), and BV (*p* = 0.071).

### 3.3. Growth Performance of Finishing Pigs

In the finishing phase, the initial body weights were similar among the treatments (*p* = 0.278, Table 5). Although no difference was observed in the final body weight among the treatments (*p* = 0.301), Met supplementation tended to exert a linear effect (*p* = 0.067). ADG, ADFI, and G:F were not affected by the dietary treatments (*p* = 0.632, 0.349, and 0.652, respectively).

### 3.4. Nitrogen Excretion and Digestibility in Finishing Pigs

During the finishing-phase metabolism trial, initial and final body weights, ADG, and G:F were not affected by the treatments (*p* = 0.982, 0.327, 0.258, and 0.301, respectively; Table 6). Similarly, the treatments did not affect fecal and urinary nitrogen excretion (*p* = 0.699 and 0.789, respectively). ATTD was similar among the treatment groups (*p* = 0.554), with no evidence of linear or quadratic responses to Met concentration (*p* > 0.05). Moreover, nitrogen retention and BV were not affected by the experimental diets (*p* = 0.243 and 0.416, respectively), and no linear or quadratic effects of dietary Met level were detected (*p* > 0.05).

### 3.5. Carcass Traits and Meat Quality of Finishing Pigs

**Figure 1:**
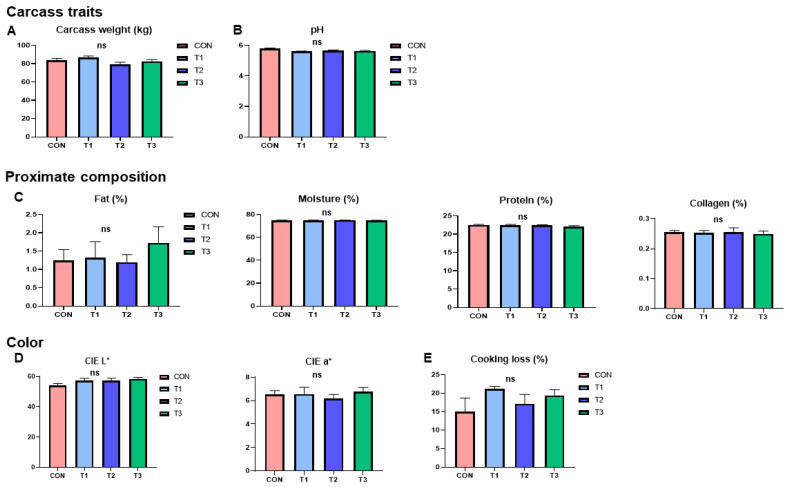
Effects of low-CP diets supplemented with graded levels of Met on carcass traits and meat quality in finishing pigs. CON = 14% CP and 0.27% Met; T1 = 13% CP and 0.27% Met; T2 = 13% CP and 0.30% Met; and T3 = 13% CP and 0.32% Met. (**A**) Carcass weight; (**B**) pH; (**C**) proximate composition of loin muscle: fat, moisture, protein, and collagen; (**D**) meat color: CIE L* (lightness) and CIE a* (redness); and (**E**) cooking loss. Values are least squares means (*n* = 5 pigs per treatment); meat quality measurements were performed in triplicate and averaged per pig. Error bars indicate standard error of the mean (SEM); ns, not significant (*p* > 0.05).

Graded Met supplementation did not affect the carcass traits and meat quality characteristics of finishing pigs (*p* > 0.05, Figure 1). Carcass weights in the CON, T1, T2, and T3 groups were 83.84 ± 1.85, 86.76 ± 1.62, 79.28 ± 2.33, and 82.62 ± 1.93 kg, respectively (Figure 1A). Although not statistically significant (*p* > 0.05), the fat content (%) was numerically higher in the T3 group than in the CON group (Figure 1C). The cooking loss rates in the CON, T1, T2, and T3 groups were 14.97 ± 3.71, 21.11 ± 0.75, 17.14 ± 2.51, and 19.40 ± 1.56%, respectively (Figure 1E).

### 3.6. Blood Biochemical Parameters

**Figure 2:**
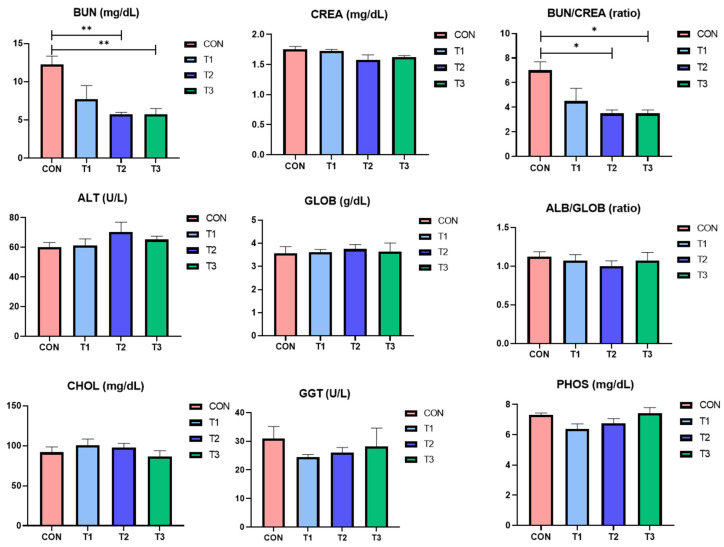
Effects of low-CP diets supplemented with graded levels of Met on blood biochemical profiles in growing pigs. CON = 16% CP and 0.37% Met; T1 = 15% CP and 0.37% Met; T2 = 15% CP and 0.41% Met; T3 = 15% CP and 0.44% Met. ALB, albumin; ALT, alanine aminotransferase; BUN, blood urea nitrogen; CHOL, total cholesterol; CP, crude protein; CREA, creatinine; GGT, gamma-glutamyl transferase; GLOB, globulin; Met, methionine; PHOS, inorganic phosphorus. ALB/GLOB indicates the albumin-to-globulin ratio. Units are mg/dL for BUN, CREA, CHOL, and PHOS; U/L for ALT and GGT; and g/dL for GLOB. BUN/CREA and ALB/GLOB are expressed as ratios. Values are presented as least-squares means ± SEM (*n* = 4 pigs per treatment). Data were analyzed using the MIXED procedure of SAS, with treatment as a fixed effect, and means were compared using Tukey’s adjustment. * *p* < 0.05; ** *p* < 0.01.

Figure 2 shows the blood biochemical parameters of growing pigs fed the experimental diets. Serum BUN levels were higher in the CON group (12.25 ± 1.11, *p* = 0.004) than in the T2 (5.75 ± 0.25, *p* < 0.01) and T3 (5.75 ± 0.75, *p* < 0.01) groups. However, there was no difference (*p* = 0.059) in serum BUN levels between the T1 (7.75 ± 1.75) and CON groups. The BUN/CREA ratio was higher in the CON group (7.00 ± 0.71, *p* = 0.013) than in the T2 (3.50 ± 0.29, *p* < 0.05) and T3 (3.50 ± 0.29, *p* < 0.05) groups. Serum CREA, ALT, GLOB, CHOL, GGT, and PHOS levels and ALB/GLOB ratios were not affected by the treatments (*p* > 0.05).

**Figure 3:**
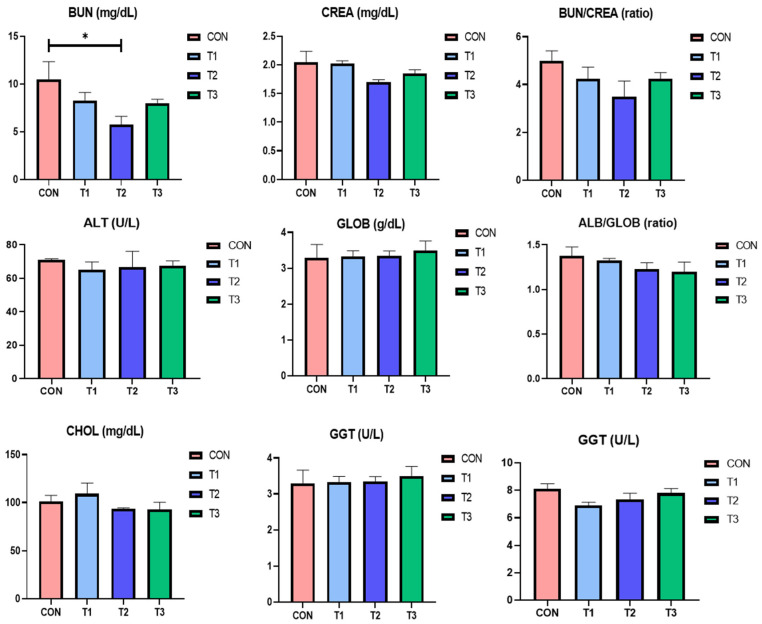
Effects of low-CP diets supplemented with graded levels of Met on blood biochemical profiles in finishing pigs. CON = 14% CP and 0.27% Met; T1 = 13% CP and 0.27% Met; T2 = 13% CP and 0.30% Met; T3 = 13% CP and 0.32% Met. ALB, albumin; ALT, alanine aminotransferase; BUN, blood urea nitrogen; CHOL, total cholesterol; CP, crude protein; CREA, creatinine; GGT, gamma-glutamyl transferase; GLOB, globulin; Met, methionine; PHOS, inorganic phosphorus. ALB/GLOB indicates the albumin-to-globulin ratio. Units are mg/dL for BUN, CREA, CHOL, and PHOS; U/L for ALT and GGT; and g/dL for GLOB. BUN/CREA and ALB/GLOB are expressed as ratios. Values are presented as least-squares means ± SEM (*n* = 4 pigs per treatment). * *p* < 0.05.

Figure 3 shows the blood biochemical parameters of finishing pigs fed the experimental diets. Serum BUN level was lower in the T2 group (5.75 ± 0.85, *p* = 0.048) than in the CON group (10.50 ± 1.85, *p* < 0.05). However, serum BUN levels did not differ between the T1 (8.25 ± 0.85), T3 (8.00 ± 0.41), and CON groups. Similarly, the CREA, ALT, GLOB, CHOL, GGT, and PHOS levels as well as BUN/CREA and ALB/GLOB ratios were not affected by the treatments (*p* > 0.05).

### 3.7. Changes in Serum Metabolic Profiles of Finishing Pigs

**Figure 4:**
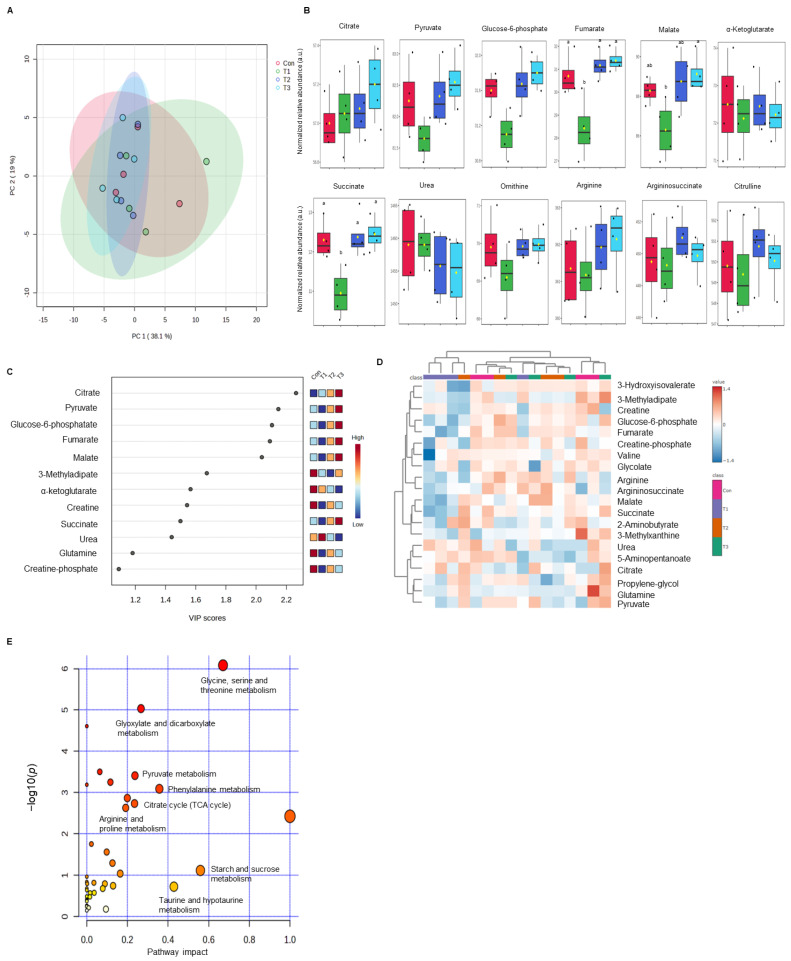
Serum metabolomic profiles of finishing pigs fed low-CP diets supplemented with graded levels of Met. CON = 14% CP and 0.27% Met; T1 = 13% CP and 0.27% Met; T2 = 13% CP and 0.30% Met; T3 = 13% CP and 0.32% Met. Serum metabolites were analyzed using untargeted ^1^H-NMR at the end of the feeding trial (*n* = 4 pigs per treatment). (**A**) Principal component analysis (PCA) score plot; (**B**) selected metabolites differing among treatments based on one-way ANOVA followed by Tukey’s multiple comparison test, expressed as normalized relative abundance (a.u.); (**C**) metabolites contributing to the exploratory multivariate pattern on variable importance in projection (VIP) scores ≥ 1.20; (**D**) heatmap of selected metabolites; and (**E**) KEGG pathway enrichment analysis. In panel (**C**), data are shown as boxplots of normalized relative abundance. Different letters in panel (**B**) indicate significant differences among treatments within each metabolite (*p* < 0.05). In panel (**E**), dot size represents pathway impact, and dot color represents the statistical significance of pathway enrichment.

Serum samples collected from four pigs per treatment at the end of the finishing phase were used for untargeted ^1^H-NMR-based metabolomic analysis. In the PCA, PC1 and PC2 explained 38.1% and 19.0% of the total variance, respectively (Figure 4A). The PCA plot showed substantial overlap among the four treatments (CON, T1, T2, and T3), with no clear separation. Because of the limited sample size, PLS-DA was used only as an exploratory supervised analysis. The PLS-DA model was generated using two retained components, with components 1 and 2 explaining 24.8% and 18.4% of the variance, respectively. Cross-validation yielded R^2^ = 0.27232 and Q^2^ = 0.06825, indicating limited predictive ability. Therefore, the PLS-DA score plot was moved to Supplementary Figure S1 and was not used as confirmatory evidence of treatment-related metabolic separation.

In the univariate analysis, serum fumarate (*p* = 0.002), succinate (*p* = 0.006), and malate (*p* = 0.028) levels differed among treatments (Figure 4B). In contrast, the serum levels of urea cycle-related metabolites, including urea, ornithine, arginine, argininosuccinate, and citrulline, were not affected by the treatments (*p* > 0.05).

Metabolites with VIP scores ≥ 1.20 were considered exploratory contributors to the multivariate pattern and included metabolites related to central carbon and energy metabolism, such as citrate (VIP = 2.06), pyruvate (VIP = 1.96), glucose-6-phosphate (VIP = 1.92), fumarate (VIP = 1.91), malate (VIP = 1.86), and α-ketoglutarate (VIP = 1.43; Figure 4C). Other VIP-selected metabolites included urea cycle-related metabolites, such as creatine (VIP = 1.41) and urea (VIP = 1.31), and amino acid metabolism-related metabolites, including glutamine (VIP = 1.20) and 3-methyladipate (VIP = 1.53). Visual inspection of the heatmap suggested that the T1 group had relatively lower intensities for some metabolites, including those related to the TCA cycle, than the T2 and T3 groups (Figure 4D). However, these heatmap patterns were described only descriptively because of the exploratory nature of the metabolomic analysis.

KEGG pathway analysis suggested several pathways with significant raw *p*-values and/or relatively high pathway impacts (Figure 4E). Among the pathways that remained significant after FDR correction and were highlighted in the Results, glycine, serine, and threonine metabolism (raw *p* = 8.25 × 10^−7^, FDR = 6.60 × 10^−5^, impact = 0.6704), phenylalanine metabolism (raw *p* = 8.13 × 10^−4^, FDR = 0.0081, impact = 0.3571), glyoxylate and dicarboxylate metabolism (raw *p* = 9.27 × 10^−6^, FDR = 0.0004, impact = 0.2667), pyruvate metabolism (raw *p* = 3.90 × 10^−4^, FDR = 0.0062, impact = 0.2370), and the citrate cycle (TCA cycle; raw *p* = 0.0018, FDR = 0.0148, impact = 0.2349) were retained as biologically relevant pathways for interpretation. Starch and sucrose metabolism showed a relatively high pathway impact but did not remain significant after FDR correction (raw *p* = 0.0763, FDR = 0.3817, impact = 0.5592). The complete KEGG pathway results, including pathways not highlighted in the main text, are provided in Supplementary Table S2.

## 4. Discussion

Recently, low-CP diets have been investigated and increasingly adopted as a nutritional strategy to reduce fecal N excretion. However, excessive reductions in CP can limit the supply of essential and nonessential amino acids, thereby impairing growth performance, including ADG and G:F. Several low-CP feeding programs have been proposed based on the ideal protein concept, in which limiting amino acids are supplemented with crystalline amino acids to maintain amino acid balance [14,15]. Low-CP diets have been shown to reduce N excretion and lower feed costs. However, optimal CP levels and responses are not consistent across studies, and the effects of low-CP diets can vary depending on diet formulation and production conditions. Therefore, the interpretation of previous findings should consider not only the absolute CP level but also the growth stage, ingredient composition, energy–amino acid balance, and environmental conditions [16].

The present study evaluated the effects of reduced dietary CP combined with Met supplementation on growth performance, carcass and meat quality traits, N utilization, and metabolic responses in pigs (growing: 16% → 15% CP; finishing: 14% → 13% CP). All experimental diets were formulated to meet the essential amino acid requirements, including lysine, and the effects of Met supplementation were evaluated as a secondary limiting amino acid response. The digestible protein (DP) content, calculated using ingredient-specific standardized ileal digestibility coefficients from the National Research Council [9], in the CON (CP, 14%) and low-CP (T1–T3; CP, 13%) groups was 11.7% and 10.9%, respectively. Although these values were slightly lower than those suggested by Rostagno et al. [16] (DP 11.31%), they were higher than those reported by Li et al. [17] (DP 9.55%), reflecting adequate overlap between the present and previous diet formulations. Under low-CP conditions, meeting the requirements for limiting amino acids is a key determinant of performance. Notably, the National Research Council [9] framework partitions amino acid requirements into maintenance and growth components, with growth requirements derived from protein deposition (PD) and utilization efficiency. In addition, given that protein deposition per daily gain (PD/DG) in finishing pigs > 80 kg falls within a relatively narrow range (14–16%) [18], the Met levels used in this study (growing: 0.37%; finishing: 0.27%, with graded supplementation) likely met the requirements of the framework [9]. The one-percentage-point CP reduction with graded Met supplementation did not significantly impair most growth performance variables during the growing and finishing phases. However, in growing pigs, ADG showed a tendency for an overall treatment effect (*p* = 0.093). Although ADG was numerically lower in the low-CP treatments than in the CON group, the response did not show a clear stepwise decline with increasing Met supplementation. Therefore, this finding should be interpreted cautiously. Consistent with previous reports, growth performance can be maintained under low-CP diets when amino acid requirements are met [15,19,20]. A meta-analysis suggested minimum CP levels of 11.6% for ADG and 11.4% for G:F in finishing pigs, indicating that 13% CP is within the acceptable range [21]. However, low-CP responses may differ across growth stages and feeding periods. For example, a previously reported 2% decrease in CP during early finishing (75–100 kg) decreased ADG [22], suggesting that sensitivity may increase with increasing CP reduction or during specific stages. Therefore, the limited performance changes observed in this study can be reasonably interpreted in the context of modest CP reduction combined with amino acid supplementation. In growing pigs, G:F showed a quadratic response to graded Met supplementation. However, this response did not indicate a consistent improvement in feed efficiency with increasing Met supplementation, as no linear response was observed. Moreover, the numerical G:F values were similar across treatments, indicating that the statistically significant quadratic effect had limited biological interpretability given its small magnitude. This may suggest that the basal Met level in the low-CP diet was sufficient to support feed efficiency, or that responses to additional Met were limited by other nutritional factors, such as overall amino acid balance. Therefore, the quadratic G:F response was considered a statistical pattern rather than clear evidence of a biologically meaningful improvement in feed efficiency. Because the feeding trials were conducted with four replicate pens per treatment, the statistical power to detect small to moderate treatment effects may have been limited. Therefore, nonsignificant growth performance responses should be interpreted cautiously within the replication level and experimental conditions of this study.

Met is a sulfur-containing essential amino acid and is considered a secondary limiting amino acid in corn–soybean meal-based diets. Generally, reduction in dietary Met in low-CP diets may exacerbate Met limitation [23]. In addition to serving as a substrate for protein synthesis (lean tissue accretion), Met participates in methyl group transfer via S-adenosylmethionine and supports antioxidant defense through trans-sulphuration pathways that contribute to cysteine and glutathione synthesis [24]. However, the absence of a growth response in this study suggests that amino acid supply (including Met) in the basal diet was sufficient for growth or that graded Met supplementation did not translate into measurable differences in growth performance. Additional supplementation may not affect growth performance when limiting amino acids are adequately supplied, and responses may instead be affected by ingredient composition, ambient temperature, and production environment [25]. These findings provide context for interpreting the limited growth response to graded Met supplementation.

Regarding carcass and meat quality, some studies have shown that low-CP diets increase fat deposition, potentially due to reduced energy costs for N excretion and altered nutrient utilization. Moreover, surplus energy may be diverted toward lipid synthesis when protein accretion is constrained [26]. Low-CP diets may promote cereal inclusion and dietary starch content, which supports lipogenesis more efficiently than protein [27]. In the present study, one-percentage-point CP reduction combined with graded Met supplementation did not significantly affect key meat quality indicators, including loin fat content and cooking loss. Low-CP diets with amino acid supplementation are expected to exert minimal effects on major carcass and meat quality traits [28]. Because carcass and meat quality traits were evaluated using a limited number of pigs per treatment, the statistical power to detect moderate differences may have been limited. Therefore, nonsignificant numerical differences in carcass weight and cooking loss should be interpreted cautiously.

The principal goal of low-CP diets is to reduce fecal N excretion. In the present study, a decrease in CP content combined with Met supplementation did not significantly affect fecal/urinary N excretion and ATTD. Although each one-percentage-point reduction in CP may reduce N excretion by ~8% [29], other studies have also reported no significant differences in fecal N excretion despite CP reductions of 1.0–1.5% or ≥2%. Given the modest CP reduction of ~1%, the differences in N utilization and excretion patterns may have been negligible. Moreover, adjustments in feed ingredients to reduce CP may alter the supply of utilizable proteins and amino acids [30]. For example, reducing high-quality protein ingredients, such as soybean meal, or increasing ingredients with lower protein availability (wheat bran) could limit N utilization even under lysine and Met supplementation owing to limited nonessential amino acid supply or overall amino acid balance [31]. In the growing-phase metabolism trial, urinary N excretion tended to increase, whereas retained N and BV tended to decrease with increasing Met supplementation among the low-CP treatments. This pattern suggests that additional Met did not improve N retention efficiency under the present low-CP condition. Excess Met may have been catabolized rather than used for protein deposition, particularly if other amino acids or nonessential nitrogen became relatively limiting. Similar responses have been reported in growing pigs, in which amino acid interactions affected N retention and amino acid utilization [32]. However, because these responses were only tendencies and ADG and G:F numerically increased from T1 to T3, this pattern was considered a possible shift in N partitioning rather than clear evidence of a negative effect of Met supplementation. In addition, the absence of linear or quadratic responses in N digestibility/utilization despite increasing Met levels suggests that supplementation with essential amino acids alone may not be sufficient and that nonessential amino acids and overall amino acid balance should be considered in low-CP diet design to maintain N utilization and minimize environmental N loss.

BUN is widely used as an indicator of amino acid deamination and urea synthesis. A decrease in CP, combined with supplementation with crystalline amino acids, has been shown to decrease BUN levels [33]. In growing pigs, serum BUN level and BUN/CREA ratio were lower in the low-CP groups (15% CP), particularly in the T2 (0.41% Met) and T3 (0.44% Met) groups, than in the CON group (16% CP). These results suggest reduced circulating urea formation in selected low-CP treatments with Met supplementation. Because CREA levels did not differ among treatments, changes in BUN and BUN/CREA were more likely attributable to altered urea formation than to impaired renal function [34]. In addition, the lack of treatment effects on ALT and GGT levels (liver-related indices), GLOB levels and ALB/GLOB ratios (protein status-related indices), and CHOL and PHOS levels suggested that the intake of low-CP diets combined with Met supplementation does not adversely affect systemic biochemical indices in growing pigs.

In finishing pigs, BUN levels differed among the treatments (decreased in one treatment relative to CON), but the magnitude of change was less than that in growing pigs. This may be related to the lower protein deposition rate and amino acid demand during the finishing phase, which could reduce the responsiveness of circulating urea-related indices. Given that N utilization (fecal/urinary N) showed minimal responses to the treatments, the observed changes in BUN levels may not necessarily translate into consistent differences in N excretion under conditions where dietary nutrient supply largely meets requirements and CP reduction is modest [35,36].

Serum metabolomic analysis was conducted as an exploratory approach to evaluate potential changes in circulating metabolite patterns in finishing pigs fed low-CP diets with graded Met supplementation. PCA showed substantial overlap among treatments, indicating no clear unsupervised separation among dietary treatments. PLS-DA was also performed as an exploratory supervised analysis; however, the low Q^2^ value indicated limited predictive ability and a potential risk of overfitting due to the small sample size. Therefore, the PLS-DA score plot was moved to the Supplementary Material and was not used as confirmatory evidence of treatment-related metabolic separation. Accordingly, the interpretation of the metabolomic data was focused primarily on univariate metabolite differences, VIP-ranked metabolites, and pathway-level patterns. Univariate analysis showed differences in selected TCA cycle-related metabolites, including fumarate, succinate, and malate, and KEGG pathway analysis suggested possible involvement of pyruvate metabolism and the citrate cycle [37]. In addition, the enrichment of glycine, serine and threonine metabolism and phenylalanine metabolism may indicate exploratory alterations in circulating amino acid-related metabolic patterns. Although phenylalanine, tyrosine, and tryptophan biosynthesis appeared in the pathway plot, this pathway was not biologically interpreted because its relevance to circulating serum metabolite changes in pigs under the present dietary conditions was limited. Moreover, because these pathway-level signals were not supported by consistent treatment effects in the corresponding individual amino acid-related metabolites, they were not considered direct evidence of altered amino acid metabolism. These findings should also be interpreted cautiously because the metabolomic analysis was based on serum samples collected at a single time point from four pigs per treatment. Serum metabolite profiles reflect systemic circulating metabolites and do not necessarily represent tissue-specific metabolic activity. Previous studies reported changes in pyruvate-related metabolites and TCA intermediates under different nutritional and physiological conditions, and Met-related perturbations were associated with changes in central carbon metabolites in serum and plasma [36,38]. Therefore, the observed differences in TCA cycle- and pyruvate-related metabolites should be regarded as exploratory changes in circulating metabolite patterns rather than direct evidence of metabolic adjustments required to maintain the TCA intermediate pool under low-CP conditions [38].

Although urea and creatine were identified as VIP-selected metabolites, they were not significant in the univariate analysis. This apparent discrepancy reflects the difference between multivariate and univariate approaches, because VIP scores indicate contribution to the overall multivariate pattern, whereas ANOVA evaluates individual metabolite differences among treatments. Therefore, urea and creatine should be interpreted as exploratory contributors to the multivariate metabolomic pattern rather than as individually altered metabolites. The absence of significant univariate changes in urea cycle-related metabolites, including urea, ornithine, arginine, argininosuccinate, and citrulline, is consistent with the modest treatment responses observed for N utilization and blood biochemical indices [39]. Glutamine and 3-methyladipate were also identified among the VIP-selected metabolites, indicating exploratory changes in circulating amino acid-related metabolite patterns under low-CP diets with graded Met supplementation. Previous research has shown that reduced dietary protein levels or alterations in amino acid profiles may affect serum nitrogen-related markers, such as N-acetylglutamate, and that reduced protein supply may lower ammonia availability for urea synthesis, complicating the interpretation of isolated marker changes as evidence of altered urea cycle activity [35]. Accordingly, the limited changes in urea cycle-related metabolites were consistent with the limited treatment responses observed for N utilization and blood indices. Glutamine is metabolically linked to the TCA cycle through conversion to α-ketoglutarate, indicating a potential connection between amino acid-related metabolites and energy metabolism even when nitrogen indices show limited variation [40]. In addition, 3-methyladipate is associated with organic acid and energy metabolism-related catabolic pathways, supporting the exploratory interpretation that low-CP diets with graded Met supplementation were associated with selected amino acid- and organic acid-related circulating metabolite patterns.

In summary, serum metabolomic analysis suggested treatment-related differences in metabolites associated with energy metabolism, particularly those related to the TCA cycle and pyruvate metabolism, in finishing pigs fed low-CP diets with graded Met supplementation. In contrast, changes in urea cycle-related metabolites were limited, consistent with the modest treatment effects observed for N utilization and blood indices [41]. Given the small sample size (*n* = 4 per treatment), the PLS-DA result was treated only as exploratory supplementary information, and further studies with larger sample sizes and broader dietary contrasts are warranted to confirm the reproducibility and biological significance of the observed metabolomic profiles.

## 5. Conclusions

A one-percentage-point reduction in dietary CP combined with graded Met supplementation did not significantly impair growth performance, carcass traits, meat quality, fecal and urinary nitrogen excretion, or ATTD of nitrogen in growing and finishing pigs under the conditions of this study. Some low-CP treatments reduced serum BUN concentrations and BUN/CREA ratios in growing pigs, suggesting potential changes in nitrogen-related biochemical responses. Serum metabolomic analysis in finishing pigs provided exploratory evidence of changes in circulating metabolites associated with energy-related pathways, including pyruvate metabolism. However, the metabolomic findings should be interpreted cautiously because they were based on serum samples collected at a single time point from a limited number of pigs. Overall, these results suggest that a modest reduction in dietary CP with graded Met supplementation may be feasible without clear adverse effects on production performance, while providing useful preliminary information for future studies on nitrogen utilization and metabolic responses under low-CP feeding conditions.

## Figures and Tables

**Table 1:** Formulations for low-crude protein (CP) diets supplemented with graded concentrations of methionine (Met) for growing pigs.

Item ^(1)^	Treatment			
	CON	T1	T2	T3
Feedstuff, %				
Corn	59.29	61.50	61.50	61.49
Soybean meal (44% crude protein)	19.00	15.50	15.48	15.47
Wheat	15.73	16.67	16.68	16.69
Soybean oil	2.53	2.65	2.62	2.60
Threonine	0.23	0.28	0.28	0.28
L-lysine	0.60	0.71	0.71	0.71
DL-methionine	0.13	0.15	0.19	0.22
Dicalcium phosphate	0.84	0.89	0.89	0.89
Limestone (CaCO_3_)	0.85	0.85	0.85	0.85
Vitamin–mineral premix	0.50	0.50	0.50	0.50
NaCl	0.30	0.30	0.30	0.30
Calculated composition				
Metabolizable energy, kcal/kg	3300	3300	3300	3300
Crude protein, %	16.00	15.00	15.00	15.00
Lysine, %	1.22	1.22	1.22	1.22
Methionine, %	0.37	0.37	0.41	0.44
Threonine, %	0.79	0.79	0.79	0.79
Ca, %	0.59	0.59	0.59	0.59
Total P, %	0.51	0.51	0.51	0.51
Analyzed composition				
Crude protein, %	16.54	15.32	15.28	15.16
Lysine, %	1.20	1.27	1.24	1.25
Methionine, %	0.39	0.40	0.42	0.44

^(1)^ The following quantities per kilogram of complete diet were provided: 10,000 IU vitamin A, 2000 IU vitamin D_3_, 12.5 IU vitamin E, 0.50 mg vitamin K_3_ (as menadione), 1.50 mg vitamin B_2_ (as riboflavin), 5.0 mg pantothenic acid (as calcium pantothenate), 10.0 mg niacin (as nicotinic acid), and 0.10 mg biotin. The diet included 60 mg Mn, 75 mg Zn, 20 mg Fe, 1.25 mg I, 0.50 mg Co, and 5.00 mg Cu.

**Table 2:** Formulations for low-CP diets supplemented with graded concentrations of Met for finishing pigs.

Item ^(1)^	Treatment			
	CON	T1	T2	T3
Feedstuff, %				
Corn	70.35	73.97	73.92	73.97
Soybean meal (44% crude protein)	11.44	9.15	9.12	9.10
Wheat	10.00	10.02	10.00	10.00
Soybean oil	4.93	3.38	3.41	3.39
Threonine	0.08	0.14	0.14	0.14
L-lysine	0.57	0.64	0.64	0.64
DL-methionine	0.10	0.11	0.14	0.16
Dicalcium phosphate	1.03	1.09	1.13	1.10
Limestone (CaCO_3_)	0.50	0.50	0.50	0.50
Vitamin–mineral premix	0.50	0.50	0.50	0.50
NaCl	0.50	0.50	0.50	0.50
Calculated composition				
Metabolizable energy, kcal/kg	3300	3300	3300	3301
Crude protein, %	14.00	13.00	13.00	13.00
Lysine, %	0.91	0.91	0.91	0.91
Methionine, %	0.27	0.27	0.30	0.32
Threonine, %	0.59	0.59	0.59	0.59
Ca, %	0.48	0.49	0.49	0.49
Total P, %	0.43	0.43	0.44	0.43
Analyzed composition				
Crude protein, %	14.35	13.22	13.26	13.37
Lysine, %	1.02	1.01	1.03	1.02
Methionine, %	0.29	0.29	0.31	0.33

^(1)^ The following quantities per kilogram of complete diet were provided: 10,000 IU vitamin A, 2000 IU vitamin D_3_, 12.5 IU vitamin E, 0.50 mg vitamin K_3_ (as menadione), 1.50 mg vitamin B_2_ (as riboflavin), 5.0 mg pantothenic acid (as calcium pantothenate), 10.0 mg niacin (as nicotinic acid), and 0.10 mg biotin. The diet included 60 mg Mn, 75 mg Zn, 20 mg Fe, 1.25 mg I, 0.50 mg Co, and 5.00 mg Cu.

**Table 3:** Growth performance of growing pigs fed low-CP diets supplemented with graded levels of Met.

Item ^(2)^	Treatment ^(1)^				SEM	*p*-Value		
	CON	T1	T2	T3		Treatment	Methionine Level	
							Linear	Quadratic
Initial BW (kg)	33.00	32.30	32.50	32.20	1.05	0.660	0.841	0.312
Final BW (kg)	60.40	57.20	58.10	59.40	1.72	0.278	0.248	0.924
ADG (kg/d)	0.64	0.58	0.60	0.63	0.05	0.093	0.106	0.843
ADFI (kg/d)	2.702	2.643	2.690	2.703	0.06	0.631	0.344	0.966
G:F	0.24	0.22	0.22	0.23	0.01	0.071	0.294	0.049

^(1)^ CON = 16% CP and 0.37% Met; T1 = 15% CP and 0.37% Met; T2 = 15% CP and 0.41% Met; T3 = 15% CP and 0.44% Met. ^(2)^ BW, body weight; ADG, average daily gain; ADFI, average daily feed intake; G:F, gain-to-feed ratio.

**Table 4:** Effects of graded Met supplementation in low-CP diets on metabolic responses in growing pigs.

Item ^(2)^	Treatment ^(1)^				SEM	*p*-Value		
	CON	T1	T2	T3		Treatment	Met Level	
							Linear	Quadratic
Growth performance								
Initial BW (kg)	62.30	62.80	61.00	61.60	2.57	0.941	0.573	0.698
Final BW (kg)	70.30	68.40	67.70	70.00	2.14	0.737	0.801	0.563
ADG (kg/d)	1.14	0.81	0.96	1.20	0.17	0.265	0.085	0.779
Feed intake (kg/d)	2.00	2.00	2.00	2.00	NA	NA	NA	NA
G:F	0.57	0.41	0.48	0.60	0.09	0.295	0.085	0.779
Nitrogen excretion								
Fecal N excreted (kg/d)	0.007	0.007	0.008	0.007	0.001	0.792	0.980	0.417
Urinary N excreted (kg/d)	0.013	0.010	0.013	0.014	0.003	0.248	0.063	0.501
ATTD of N (%)	84.624	84.501	82.796	84.444	2.67	0.749	0.980	0.417
Retained N (kg/d)	0.027	0.027	0.023	0.023	0.002	0.212	0.079	0.321
BV (%)	67.182	73.441	64.553	62.222	6.90	0.286	0.071	0.514

^(1)^ CON = 16% CP and 0.37% Met; T1 = 15% CP and 0.37% Met; T2 = 15% CP and 0.41% Met; T3 = 15% CP and 0.44% Met. ^(2)^ BW, body weight; ADG, average daily gain; G:F, gain-to-feed ratio; ATTD, apparent total tract digestibility; Retained N, nitrogen consumed minus nitrogen in feces and urine; BV, biological value; NA, not applicable. Feed intake was fixed according to the metabolism trial feeding regimen. Pigs were fed at 3% of average body weight in two equal meals per day; therefore, SEM and *p*-values for feed intake were not calculated.

**Table 5:** Growth performance of finishing pigs fed low-CP diets supplemented with graded levels of Met.

Item ^(2)^	Treatment ^(1)^				SEM	*p*-Value		
	CON	T1	T2	T3		Treatment	Met Level	
							Linear	Quadratic
Initial BW (kg)	60.40	57.15	58.10	59.35	1.72	0.278	0.152	0.189
Final BW (kg)	110.00	105.15	105.00	107.25	2.25	0.301	0.067	0.939
ADG (kg/d)	0.99	0.96	0.94	0.96	0.05	0.632	0.434	0.918
ADFI (kg/d)	3.741	3.659	3.736	3.737	0.03	0.349	0.199	0.471
G:F	0.26	0.26	0.25	0.26	0.01	0.652	0.383	0.457

^(1)^ CON = 14% CP and 0.27% Met; T1 = 13% CP and 0.27% Met; T2 = 13% CP and 0.30% Met; T3 = 13% CP and 0.32% Met. ^(2)^ BW, body weight; ADG, average daily gain; ADFI, average daily feed intake; G:F, gain-to-feed ratio.

**Table 6:** Effects of graded Met supplementation in low-CP diets on metabolic responses in finishing pigs.

Item ^(2)^	Treatment ^(1)^				SEM	*p*-Value		
	CON	T1	T2	T3		Treatment	Met Level	
							Linear	Quadratic
Growth performance								
Initial BW (kg)	88.17	87.83	87.25	87.83	1.622	0.982	1.000	0.790
Final BW (kg)	94.92	93.92	93.50	94.50	1.652	0.327	0.819	0.749
ADG (kg/d)	0.96	0.87	0.89	0.95	0.103	0.258	0.531	0.876
Feed intake (kg/d)	2.60	2.60	2.60	2.60	.	.	.	.
G:F	0.37	0.33	0.34	0.37	0.041	0.301	0.532	0.876
Nitrogen excretion								
Fecal N excreted (kg/d)	0.027	0.028	0.030	0.026	0.002	0.699	0.805	0.698
Urinary N excreted (kg/d)	0.006	0.006	0.008	0.008	0.001	0.789	0.665	0.828
ATTD of N (%)	68.542	63.643	59.684	66.853	5.995	0.554	0.713	0.570
Retained N (kg/d)	0.018	0.015	0.012	0.015	0.003	0.243	0.844	0.500
BV (%)	75.524	66.791	54.982	63.373	12.114	0.416	0.971	0.515

^(1)^ CON = 14% CP and 0.27% Met; T1 = 13% CP and 0.27% Met; T2 = 13% CP and 0.30% Met; T3 = 13% CP and 0.32% Met. ^(2)^ BW, body weight; ADG, average daily gain; G:F, gain-to-feed ratio; ATTD, apparent total tract digestibility; Retained N, nitrogen consumed minus nitrogen in feces and urine; BV, biological value; Feed intake was fixed according to the metabolism trial feeding regimen. Pigs were fed at 3% of average body weight in two equal meals per day; therefore, SEM and *p*-values for feed intake were not calculated.

## Data Availability

The raw data supporting the conclusions of this article will be made available by the authors on request.

## Supplementary Materials

The following supporting information can be downloaded at: https://www.mdpi.com/article/10.3390/ani16111687/s1. Table S1. Treatment allocation matrix for the metabolism trials. Table S2. KEGG pathway statistics from serum metabolomic pathway analysis. Figure S1. Exploratory PLS-DA score plot of serum metabolomic profiles in finishing pigs fed low-CP diets supplemented with graded levels of Met.

## Abbreviations

The following abbreviations are used in this manuscript:

CP	Crude protein
ADG	Average daily gain
G:F	Gain-to-feed ratio
ADFI	Average daily feed intake
ATTD	Apparent total tract digestibility
PCA	Principal component analysis
PLS-DA	Partial least squares–discriminant analysis
VIP	Variable importance in projection
KEGG	Kyoto Encyclopedia of Genes and Genomes
BV	Biological value
DP	Digestible protein
PD	Protein deposition
PD/DG	Protein deposition per daily gain
BUN	Blood urea nitrogen
CREA	Creatinine
ALB	Albumin
GLOB	Globulin
BUN/CREA	Blood urea nitrogen-to-creatinine ratio
ALB/GLOB	Albumin-to-globulin ratio
ALT	Alanine aminotransferase
GGT	Gamma-glutamyl transferase
CHOL	Total cholesterol
PHOS	Inorganic phosphorus

## References

[1] OECD/FAO (2025). OECD-FAO Agricultural Outlook 2025–2034.

[2] Wyer K.E., Kelleghan D.B., Blanes-Vidal V., Schauberger G., Curran T.P. (2022). Ammonia emissions from agriculture and their contribution to fine particulate matter: A review of implications for human health. J. Environ. Manag..

[3] Wang Y., Zhou J., Wang G., Cai S., Zeng X., Qiao S. (2018). Advances in low-protein diets for swine. J. Anim. Sci. Biotechnol..

[4] Kerr B.J., Southern L.L., Bidner T.D., Friesen K.G., Easter R.A. (2003). Influence of dietary protein level, amino acid supplementation, and dietary energy levels on growing-finishing pig performance and carcass composition. J. Anim. Sci..

[5] Panetta D.M., Powers W.J., Xin H., Kerr B.J., Stalder K.J. (2006). Nitrogen excretion and ammonia emissions from pigs fed reduced crude protein diets or yucca extract. J. Environ. Qual..

[6] Niyonsaba A., Jin X.H., Kim Y.Y. (2023). Effect of reducing dietary crude protein level on growth performance, blood profiles, nutrient digestibility, carcass traits, and odor emissions in growing-finishing pigs. Anim. Biosci..

[7] Zhao Y., Qin G., Sun Z., Zhang X., Bao N., Wang T., Zhang J. (2019). Effect of different dietary protein levels and amino acids supplementation patterns on growth performance, carcass characteristics and nitrogen excretion in growing-finishing pigs. J. Anim. Sci. Biotechnol..

[8] van Milgen J., Dourmad J.Y. (2015). Concept and application of ideal protein for pigs. J. Anim. Sci. Biotechnol..

[9] National Research Council (2012). Nutrient Requirements of Swine.

[10] Kong C., Ragland D., Adeola O. (2014). Ileal endogenous amino acid flow response to nitrogen-free diets with differing ratios of corn starch to dextrose in pigs. Asian-Australas. J. Anim. Sci..

[11] Aaron D.K., Hays V.W. (2004). How many pigs? Statistical power considerations in swine nutrition experiments. J. Anim. Sci..

[12] Kim B.G., Kim T. (2010). A program for making completely balanced Latin square designs employing a systemic method. Rev. Colomb. Cienc. Pecu..

[13] AOAC (2016). Official Methods of Analysis of AOAC International.

[14] Han I.K., Lee J.H. (2000). The role of synthetic amino acids in monogastric animal production: Review. Asian-Australas. J. Anim. Sci..

[15] Duarte M.E., Parnsen W., Zhang S., Abreu M.L.T., Kim S.W. (2024). Low crude protein formulation with supplemental amino acids for its impacts on intestinal health and growth performance of growing-finishing pigs. J. Anim. Sci. Biotechnol..

[16] Rostagno H.S., Albino L.F.T., Hannas M.I., Donzele J.L., Sakomura N.K., Perazzo F.G., Saraiva A., Teixeira M.L., Rodrigues P.B., de Oliveira R.F. (2017). Brazilian Tables for Poultry and Swine: Composition of Feedstuffs and Nutritional Requirements.

[17] Li S., Zhang X., Liu H., Wang C., Li Z., Zhang G., Wang F. (2025). Dietary digestible protein requirement in finishing pigs: A study for experimental determination and verification. Agriculture.

[18] de Lange C.F.M., Morel P.C.H., Birkett S.H. (2003). Modeling chemical and physical body composition of the growing pig. J. Anim. Sci..

[19] Ball M.E.E., Magowan E., McCracken K.J., Beattie V.E., Bradford R., Gordon F.J., Robinson M.J., Smyth S. (2013). The effect of level of crude protein and available lysine on finishing pig performance, nitrogen balance and nutrient digestibility. Asian-Australas. J. Anim. Sci..

[20] Cho I., Kong C. (2025). Growth performance of pigs fed low-protein diets supplemented with crystalline amino acids at different growth stages. Anim. Biosci..

[21] Rocha G.C., Doldan M.E., Kim S.W. (2022). Advances, implications, and limitations of low-crude-protein diets in pig production. Animals.

[22] Wang Y.M., Li F.C., Yang X.J., Li J.Q., Zhang G.J., Ding X.B., Xiong X., Yin Y.L. (2019). Effects of feeding growing-finishing pigs with low crude protein diets on growth performance, carcass characteristics, meat quality and nutrient digestibility in different areas of China. Anim. Feed. Sci. Technol..

[23] Yang Z., Htoo J.K., Liao S.F. (2020). Methionine nutrition in swine and related monogastric animals: Beyond protein biosynthesis. Anim. Feed. Sci. Technol..

[24] Brosnan J.T., Brosnan M.E. (2006). The sulfur-containing amino acids: An overview. J. Nutr..

[25] de Oliveira M.J.K., Cemin H.S., Vier C.M., Sbardella M., Bernardi M.L., Wentz I., Bortolozzo F.P., Kiefer C. (2023). Effects of lowering dietary protein content without or with increased protein-bound and feed-grade amino acids supply on growth performance, body composition, metabolism, and acute-phase protein of finishing pigs under daily cyclic heat stress. J. Anim. Sci..

[26] Tuitoek K., Young L.G., de Lange C.F.M., Kerr B.J. (1997). The effect of reducing excess dietary amino acids on growing-finishing pig performance: An evaluation of the ideal protein concept. J. Anim. Sci..

[27] van Milgen J., Noblet J., Dubois S. (2001). Energetic efficiency of starch, protein and lipid utilization in growing pigs. J. Nutr..

[28] Han Y.G., Lee G.I., Do S.H. (2023). The effect of reduced crude protein on growth performance, nutrient digestibility, and meat quality in weaning to finishing pigs. Animals.

[29] Pomar C., Remus A., Rivera-Ferre M.G. (2021). Feeding strategies to reduce nutrient losses and improve the sustainability of growing pigs. Front. Vet. Sci..

[30] Cappelaere L., Dourmad J.Y., van Milgen J., Kebreab E. (2024). Environmental benefits of crude protein reduction in growing pigs: A meta-analysis approach. J. Anim. Sci..

[31] Gloaguen M., Le Floc’h N., Corrent E., Primot Y., van Milgen J. (2014). The use of free amino acids allows formulating very low crude protein diets for piglets. J. Anim. Sci..

[32] Langer S., Scislowski P.W.D., Brown D.S., Dewey P., Fuller M.F. (2000). Interactions among the branched-chain amino acids and their effects on methionine utilization in growing pigs: Effects on nitrogen retention and amino acid utilization. Br. J. Nutr..

[33] Gómez R.S., Lewis A.J., Miller P.S., Chen H.Y. (2002). Growth performance, diet apparent digestibility, and plasma metabolites of pigs fed standard corn-soybean meal diets or low-protein diets supplemented with crystalline amino acids. J. Anim. Sci..

[34] Hosten A.O., Walker H.K., Hall W.D., Hurst J.W. (1990). BUN and creatinine. Clinical Methods: The History, Physical, and Laboratory Examinations.

[35] Kohn R.A., Dinneen M.M., Russek-Cohen E. (2005). Using blood urea nitrogen to predict nitrogen excretion and efficiency of nitrogen utilization in cattle, sheep, goats, horses, pigs, and rats. J. Anim. Sci..

[36] Spring S., Premathilake H., De Angelis M., Lallès J.P., Zähner M., Gerber P.J., Steiner T. (2020). Low protein-high carbohydrate diets alter energy balance, gut microbiota composition and blood metabolomics profile in young pigs. Sci. Rep..

[37] Kanehisa M., Goto S. (2000). KEGG: Kyoto Encyclopedia of Genes and Genomes. Nucleic Acids Res..

[38] Owen O.E., Kalhan S.C., Hanson R.W. (2002). The key role of anaplerosis and cataplerosis for citric acid cycle function. J. Biol. Chem..

[39] Sobotka W., Drażbo A. (2025). The effect of dietary protein restriction in phase feeding systems on nitrogen metabolism and excretion in pig production. Animals.

[40] Yoo H.C., Yu Y.C., Sung Y.S., Han J.M. (2020). Glutamine reliance in cell metabolism. Exp. Mol. Med..

[41] Coma J., Carrion D., Zimmerman D.R. (1995). Use of plasma urea nitrogen as a rapid response criterion to determine the lysine requirement of pigs. J. Anim. Sci..

