# Supplementary material for: Effects of Reduced Crude Protein Diets with Graded Methionine Supplementation on Growth Performance, Nitrogen Utilization, and Serum Metabolomic Profiles in Growing–Finishing Pigs
Source: Animals (Basel). 2026 May 31;16(11):1687. doi: 10.3390/ani16111687 (PMC13255844; doi:10.3390/ani16111687)
Supplement: Supplementary file 1 [file animals-16-01687-s001.zip › Supplementary_Figure_S1_Exploratory PLS-DA score plot.pdf]

Supplementary Materials

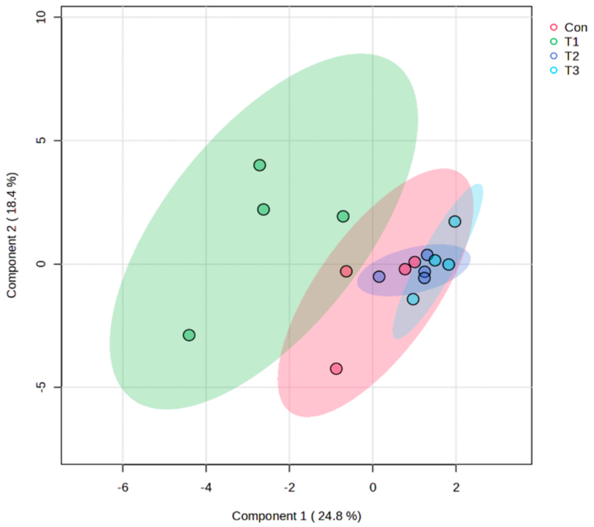

Supplementary Figure S1. Exploratory PLS-DA score plot of serum metabolomic profiles in finishing pigs fed low-CP diets supplemented with graded levels of Met. CON = 14% CP and 0.27% Met; T1 = 13% CP and 0.27% Met; T2 = 13% CP and 0.30% Met; and T3 = 13% CP and 0.32% Met. The PLS-DA model was generated using two retained components, with components 1 and 2 explaining 24.8% and 18.4% of the variance, respectively. Cross-validation yielded $R^2 = 0.27232$ and $Q^2 = 0.06825$. Because of the limited sample size and low Q^2 value, this plot was used only as an exploratory supervised visualization and was not interpreted as confirmatory evidence of treatment-related metabolic separation.
