# Supplementary material for: Effects of Reduced Crude Protein Diets with Graded Methionine Supplementation on Growth Performance, Nitrogen Utilization, and Serum Metabolomic Profiles in Growing–Finishing Pigs
Source: Animals (Basel). 2026 May 31;16(11):1687. doi: 10.3390/ani16111687 (PMC13255844; doi:10.3390/ani16111687)
Supplement: Supplementary file 1 [file animals-16-01687-s001.zip › Supplementary_Table S2_KEGG pathway statistics from serum metabolomic pathway analysis.pdf]

Supplementary Table S2. KEGG pathway statistics from serum metabolomic pathway analysis

This supplementary file provides processed pathway analysis results from serum ^{1}H-NMR-based metabolomic profiling of finishing pigs fed reduced-crude-protein diets with graded methionine supplementation.

Abbreviations: FDR, false discovery rate; Impact, pathway topology impact. The -log10(p) column represents the negative base-10 logarithm of the raw p-value.

Pathway	Total	Expected	Hits	Raw p	-log10(p)	Holm adjust	FDR	Impact
Phenylalanine, tyrosine and tryptophan biosynthesis	4	0.103	2	0.0038	2.4249	0.2594	0.0251	1
Glycine, serine and threonine metabolism	33	0.8499	8	8.25E-07	6.0834	6.60E-05	6.60E-05	0.6704
Starch and sucrose metabolism	18	0.4636	2	0.0763	1.1172	1	0.3817	0.5592
Taurine and hypotaurine metabolism	8	0.206	1	0.1888	0.7241	1	0.6292	0.4286
Phenylalanine metabolism	8	0.206	3	8.13E-04	3.0902	0.0593	0.0081	0.3571
Glyoxylate and dicarboxylate metabolism	32	0.8241	7	9.27E-06	5.0328	7.32E-04	3.71E-04	0.2667
Pyruvate metabolism	26	0.6696	5	3.90E-04	3.409	0.0296	0.0062	0.237
Citrate cycle (TCA cycle)	36	0.9271	5	0.0018	2.7335	0.1311	0.0148	0.2349
Arginine and proline metabolism	20	0.5151	4	0.0014	2.8656	0.0981	0.0121	0.1993
One carbon pool by folate	23	0.5923	4	0.0024	2.6281	0.1648	0.0171	0.1914
Tyrosine metabolism	42	1.0817	3	0.091	1.0409	1	0.4283	0.1643
Inositol phosphate metabolism	30	0.7726	2	0.1794	0.7461	1	0.6241	0.1294
Cysteine and methionine metabolism	33	0.8499	3	0.0507	1.2953	1	0.2702	0.1263
Alanine, aspartate and glutamate metabolism	28	0.7211	5	5.61E-04	3.2512	0.0421	0.0074	0.1162
Glycolysis or Gluconeogenesis	26	0.6696	3	0.0273	1.5634	1	0.1562	0.0979
Tryptophan metabolism	41	1.0559	1	0.6616	0.1794	1	1	0.0942
Glutathione metabolism	28	0.7211	2	0.1609	0.7935	1	0.5851	0.0887
Vitamin B6 metabolism	9	0.2318	1	0.2098	0.6783	1	0.6454	0.0784
Arginine biosynthesis	14	0.3605	4	3.15E-04	3.5016	0.0243	0.0062	0.0638
Pyrimidine metabolism	39	1.0044	2	0.2657	0.5755	1	0.7393	0.0363
Galactose metabolism	27	0.6954	2	0.1518	0.8188	1	0.5851	0.035
Valine, leucine and isoleucine degradation	40	1.0302	4	0.0177	1.752	1	0.1089	0.0226
Purine metabolism	70	1.8028	3	0.268	0.5719	1	0.7393	0.0162
Primary bile acid biosynthesis	46	1.1847	2	0.3335	0.4769	1	0.8338	0.0152
Glycerophospholipid metabolism	36	0.9271	1	0.6132	0.2124	1	1	0.0094
Lipoic acid metabolism	28	0.7211	2	0.1609	0.7935	1	0.5851	0.0017
Valine, leucine and isoleucine biosynthesis	8	0.206	4	2.47E-05	4.608	0.0019	6.58E-04	0
Neomycin, kanamycin and gentamicin biosynthesis	2	0.0515	2	6.47E-04	3.1888	0.0479	0.0074	0
Propanoate metabolism	22	0.5666	2	0.1081	0.9661	1	0.4806	0
Nitrogen metabolism	6	0.1545	1	0.1451	0.8383	1	0.5851	0
Ascorbate and aldarate metabolism	9	0.2318	1	0.2098	0.6783	1	0.6454	0
Caffeine metabolism	10	0.2575	1	0.2302	0.6378	1	0.6822	0
D-Amino acid metabolism	15	0.3863	1	0.3251	0.488	1	0.8338	0
Butanoate metabolism	15	0.3863	1	0.3251	0.488	1	0.8338	0
Ubiquinone and other terpenoid-quinone biosynthesis	19	0.4893	1	0.3926	0.406	1	0.9518	0
Pantothenate and CoA biosynthesis	20	0.5151	1	0.4084	0.3889	1	0.961	0
beta-Alanine metabolism	21	0.5408	1	0.4239	0.3728	1	0.9689	0
Lysine degradation	30	0.7726	1	0.5462	0.2627	1	1	0
Porphyrin metabolism	31	0.7984	1	0.5581	0.2533	1	1	0
Sphingolipid metabolism	32	0.8241	1	0.5697	0.2444	1	1	0
Fatty acid biosynthesis	47	1.2104	1	0.7119	0.1475	1	1	0

Note: Values are provided as processed pathway analysis outputs for supplementary reporting.
