# Supplementary material for: Effects of Reduced Crude Protein Diets with Graded Methionine Supplementation on Growth Performance, Nitrogen Utilization, and Serum Metabolomic Profiles in Growing–Finishing Pigs
Source: Animals (Basel). 2026 May 31;16(11):1687. doi: 10.3390/ani16111687 (PMC13255844; doi:10.3390/ani16111687)
Supplement: Supplementary file 1 [file animals-16-01687-s001.zip › Supplementary_Table_S1_Treatment_Allocation matrix for the metabolism trials_260509.pdf]

Supplementary Table S1. Treatment allocation matrix for the metabolism trials

Two-period balanced incomplete Latin square allocation used for both the growing- and finishing-phase metabolism trials.

Treatment code used in the allocation program

Program code	Treatment
A	CON
B	T1
C	T2
D	T3

Allocation matrix

Square	Animal	Period 1 code	Period 1 treatment	Period 2 code	Period 2 treatment
1	1	A	CON	D	T3
1	2	B	T1	A	CON
1	3	C	T2	B	T1
1	4	D	T3	C	T2
2	5	C	T2	D	T3
2	6	D	T3	B	T1
2	7	A	CON	C	T2
2	8	B	T1	A	CON
3	9	B	T1	D	T3
3	10	D	T3	A	CON
3	11	A	CON	C	T2
3	12	C	T2	B	T1

Observation summary

Item	Value
Number of treatments	4
Number of replicate squares	3
Number of animals per period	12
Observations per treatment in each period	3
Total observations per treatment across two periods	6

Notes. CON, control diet; T1-T3, low-CP diets with graded methionine supplementation. In the growing phase, CON = 16% CP and 0.37% Met; T1 = 15% CP and 0.37% Met; T2 = 15% CP and 0.41% Met; T3 = 15% CP and 0.44% Met. In the finishing phase, CON = 14% CP and 0.27% Met; T1 = 13% CP and 0.27% Met; T2 = 13% CP and 0.30% Met; T3 = 13% CP and 0.32% Met. The same allocation structure was applied to both phase-specific metabolism trials. The allocation was generated using a spreadsheet-based Latin square allocation program described by Kim and Kim (2010) to balance treatment representation across periods and reduce the possibility of carryover by avoiding repeated treatment assignment to the same animal. Reference: Kim, B.G.; Kim, T. A program for making completely balanced Latin square designs employing a systemic method. Rev. Colomb. Cienc. Pecu. 2010, 23, 277-282. https://doi.org/10.17533/udea.rccp.324588
